# Editorial: Microbial Stress: From Model Organisms to Applications in Food, Microbiotechnology and Medicine

**DOI:** 10.3389/fmicb.2022.945573

**Published:** 2022-06-27

**Authors:** Aleksandra Djukić-Vuković, Nuno P. Mira, Jana Sedlakova-Kadukova, Daniela De Biase, Peter A. Lund

**Affiliations:** ^1^Department of Biochemical Engineering and Biotechnology, Faculty of Technology and Metallurgy, University of Belgrade, Belgrade, Serbia; ^2^Institute for Bioengineering and Biosciences, Instituto Superior Técnico—Department of Bioengineering, Universidade de Lisboa, Lisboa, Portugal; ^3^Associate Laboratory i4HB—Institute for Health and Bioeconomy at Instituto Superior Técnico, Universidade de Lisboa, Lisboa, Portugal; ^4^Department of Ecochemistry and Radioecology, Faculty of Natural Sciences, University of St. Cyril and Methodius in Trnava, Trnava, Slovakia; ^5^Department of Medico-Surgical Sciences and Biotechnologies, Sapienza University of Rome, Latina, Italy; ^6^Institute of Microbiology and Infection, School of Biosciences, University of Birmingham, Birmingham, United Kingdom

**Keywords:** methodologies in microbial stress studies, adaptive responses in microbial stress and tolerance, structure-function relationship in stress adaptation, acid stress, OMICS stress responses, food safety, stress responses and bioremediation, antimicrobial stress responses

Within this Research Topic (RT) we have collected articles addressing microbial stress with special attention on methodology and applications in food technology, medicine or microbiotechnology. We anticipated contributions from members of COST action CA18113 EuroMicropH “*Understanding and exploiting the impact of low pH on micro-organism*” (COST, 2019), but in the final collection (which attracted 85 authors from 17 countries), we were happy to see many contributing authors from outside this network. This shows the breadth and importance of the topic and also the potential for expansion of the COST network. With 14 original research articles, 1 methods and 1 review article, this RT shows that microbial stress in the above contexts is mostly a result of a combination of stressors. This makes it challenging to understand mechanisms and exploit this knowledge although significant efforts are dedicated to it (De Biase et al., 2020a,b; Lund et al., 2020). The main goal of our EuroMicropH community is to translate research on low pH in particular, and microbial stress in general, between fields, and to identify conceptual and methodological gaps in that knowledge. Our RT helps to address this. We hope it will help the development of new approaches to infection control, while improving food and drink processing and the use of microorganisms in green industrial processes.

## New Methodologies to Study Microorganisms Under Stress

Modeling microbial growth under stress conditions is crucial to ensure food safety. Lag phase is one important component that can be hard to model. Akkermans and Van Impe present a new method combining improved sampling to reduce error, and simulations to identify which parameters are most important in providing accurate estimates of lag phase under different conditions. Beyond food safety applications, monitoring of the growth and cellular metabolic state helps increase bioprocess productivity. To accelerate the design of high-performing yeast strains, Pianale et al. developed a toolbox of intracellular biosensors to monitor pH, redox state, ATP levels, glycolytic flux, and ribosome production, all important for a robust stress response. They demonstrated how these biosensors can be used to show how these parameters are affected by stress during fermentation of a synthetic wheat straw hydrolysate.

## Fermentation Stress

Microbe-based hydrolysis and fermentation of lignocellulose is a key target for green economies, but it can be hampered by compounds such as furfural that arise during biomass pretreatment. A furfural-resistant *Zymomonas mobilis* strain was characterized by Hu et al. using complementation and RNA-seq, and they identified several aspects of its resistance mechanism, including increased breakdown of furfural, increased flocculation, increased chaperone production, and down-regulation of energy-intensive transporters. Didak Ljubas et al. also identified furfural as an important inhibitor of *Paenibacillus polymyxa* DSM 742, a newly-used bacterial strain for the production of 2,3-butanediol, lactic acid and ethanol. Fermentation stress caused by changes in pH of media, presence of furfural and oxygen caused changes in metabolic activity which can be valorized by process optimization and strain adaptation. This should help future engineering of improved strains.

Fermentation stress is caused by combination of a stressors, and adaptive laboratory evolution is a convenient technique to study it and get more resistant strains. Resistance to fermentation stress in the acetic acid bacterium *Komagataeibacter medellinensis* was studied in this way by Kataoka et al.. Through this approach they obtained strains with improved growth and fermentation yield at high temperatures in the presence of ethanol and acetic acid. Whole genome sequencing revealed mutations in *gyrB, spoT*, and *degP*. The effects of mutation of the latter two genes alone were studied by making further targeted mutations, the analysis of which suggested they have roles in cell surface stability and cell elongation. More in depth analysis of oxidative stress, which is often seen in bioprocesses and can cause problems with yield, was provided by Kocaefe-Özşen et al.. They also used adaptive laboratory evolution, in this case to produce strains of the yeast *Saccharomyces cerevisiae* that showed enhanced resistance to oxidative stress. A full physiological and genomic characterization of the resistant strains showed many adaptations, including elevated trehalose and glycogen synthesis, and also showed the strains had become resistant to other additional stresses such as heat, high salt, and ethanol. Although these studies were performed for intensification of bioprocesses, results may also be useful in the food industry, where these stressors are applied intentionally with aim of inactivating food-borne pathogens, or in hurdle technology approaches.

## Low pH, NaCl, and Nitrosative Stress in Health and Disease

Pathogenic microorganisms are exposed to low pH or NaCl in food by means of preservation, to low pH, bile salts and varying oxygen concentration in the gastrointestinal tract (GIT), and to nitrosative stress as a defense mechanism in GIT, skin, and other mucosal surfaces. Under physiological conditions, or during infection or food processing, selective environments force microorganisms to adapt, and this will determine their abilities to colonize and survive. This is the case both with pathogens (e.g., *L. monocytogenes, S. aureus*, etc.) or with organisms that are beneficial (e.g., as probiotics) for the host.

Among food-borne pathogens, *Listeria monocytogenes* has an important place, causing even fatal outcomes. Lakićević et al. describe the mechanisms that *L. monocytogenes* can employ to persist in different habitats, such as farms, food production environments, and foods. The authors focused on the stress response genes that allow *L. monocytogenes* to grow at low pH, low temperature, high salt concentrations as well as on the virulence genes that contribute to the high degree of strain divergence. In the case of one particular strain, *L. monocytogenes* F2365, Chakravarty et al. studied transcription variations when exposed to acid and bile salts under anaerobic and aerobic conditions. Upon exposure to anaerobiosis in acidic conditions, variations in the transcript levels for the virulence factors including internalins and listeriolysin O were determined as well as for many histidine sensory kinases, helping to understand strain response to varying oxygen concentration throughout GIT.

*Brucella* species are another important group of pathogens, which can be transmitted in contaminated foods as well as *via* other routes, and acid resistance is an important factor determining the their prevalence. de la Garza-García et al. used RNAseq to compare acid responses of two species, one of which (*B. microti*) is particularly resistant to acid, and identified a wide range of responses, some in common and some unique to *B. microti*. These included induction of the cold shock protein CspA and the general stress protein Dps, together with other changes which may be relevant to the survival of these organisms in soil or foods. Although NaCl is commonly used for food preservation, it is identified as a risk factor for cardiovascular diseases and significant effort is being made to decrease its level in food. However, work of Etter et al. showed the important influence of NaCl on the expression of staphylococcal enterotoxin C (SEC), a cause of foodborne intoxication. By using qRT-PCR and analyzing protein expression levels, they found that mild NaCl stress as a hurdle can cause a decrease in SEC levels in different *Staphylococcus aureus* strains. Therefore, while lowering salt content in food is regarded as a positive way to reduce cardiovascular diseases, it could lead to an increase in SEC production and therefore food poisoning.

The neurotransmitter NO_2_ can accumulate in high concentrations and lead to nitrosative stress. Nasuno et al. described how pathogenic *Candida glabrata* cells cope with nitrosative stress through the activity of the riboflavin biosynthesis gene *RIB1*. It was also shown that expression of *RIB1* increases survival of *C. glabrata* in the presence of macrophage-like cells. Genes that, like *RIB1*, mediate tolerance of *C. glabrata* to stress are an interesting target for the development of new therapeutic targets. Gu et al. reported also potential therapeutic agents produced by *Bacillus* sp. YJ17 isolated from the deep-sea cold seep. Two compounds belonging to family of fengycin and surfactin showed stronger antibacterial properties when compared to the commercial food preservative nisin, and were efficient against multidrug-resistant bacteria. Synergies between stressors could be important for antimicrobial activity as confirmed by Masoura et al. for honey. The authors used genome-wide transposon mutagenesis combined with high-throughput sequencing (TraDIS) to identify genes in *E. coli* K-12 MG1655 which are needed for resistance to honey's antimicrobial effects. They include genes which encode membrane proteins like those involved in uptake of essential molecules, and components of the electron transport chain. They are enriched for pathways involved in intracellular homeostasis and redox activity. Moreover, Lovšin et al. obtained interesting results showing that exposure to abiotic stress by electroporation, a phenomenon mainly related to cell membranes, can modulate the susceptibility of *E. coli* to antibiotics with mechanisms which go beyond membrane permeabilization.

## Metals as Microbial Stressors in the Environment

Joudeh et al. reported that Pd-stress induced wide changes in *E. coli* genomic expression, resulting in drastic alterations in the expression of transporters of inorganic ion complexes. Additionally, up-regulation of genes encoding multidrug-efflux pumps was suggested to improve tolerance to Pd by reducing intracellular accumulation due to reduced influx and improved efflux. Other detoxification strategies included intracellular chelation of Pd by histidine and histidine-rich proteins and enzyme-mediated formation of less toxic nanoparticles. The generated knowledge is expected to contribute to the design of Pd-tolerant *E. coli* strains, accelerating microbial production of this important catalyst. Šimonovičová et al. reported on potential of *Aspergillus niger* as a Ni-bioleaching microorganism for treatment of hazardous ash waste with *Chlorella vulgaris* biomass being used for the biosorption of Ni. The total process efficiency reached 46% suggesting a new route not only for waste reduction, but also for recovery of valuable metals for industry.

## Author Contributions

AD-V, DD, NM, JS-K, and PL did literature survey, wrote the section of the manuscript, and reviewed and corrected the manuscript. AD-V prepared draft version of the manuscript. All authors agreed on the final version of the manuscript.

## Funding

This work was supported by the COST Action CA18113 and for AD-V by the Ministry of Education, Science and Technological Development of the Republic of Serbia (Contract No. 451-03-68/2022-14/200135).

## Conflict of Interest

The authors declare that the research was conducted in the absence of any commercial or financial relationships that could be construed as a potential conflict of interest.

## Publisher's Note

All claims expressed in this article are solely those of the authors and do not necessarily represent those of their affiliated organizations, or those of the publisher, the editors and the reviewers. Any product that may be evaluated in this article, or claim that may be made by its manufacturer, is not guaranteed or endorsed by the publisher.
